# Training for Rectal Resection in the Era of Total Neoadjuvant Therapy and Organ Preservation: Safeguarding Competence as Operative Casemix Evolves

**DOI:** 10.1111/ans.70679

**Published:** 2026-04-10

**Authors:** Rathin Gosavi, Stephen Bell, Satish Warrier, William Teoh, Paul McMurrick, Vignesh Narasimhan

**Affiliations:** ^1^ Department of Colorectal Surgery Cabrini Health Melbourne Victoria Australia; ^2^ Department of Colorectal Surgery Monash Health Melbourne Victoria Australia; ^3^ Department of Colorectal Surgery Alfred Health Melbourne Victoria Australia; ^4^ Department of Colorectal Surgery Peter MacCallum Cancer Centre Melbourne Victoria Australia; ^5^ Department of Surgery (School of Clinical Sciences at Monash Health) Monash University Melbourne Victoria Australia

The management of rectal cancer has changed substantially over the past decade. Total neoadjuvant therapy (TNT), response‐adapted protocols and structured watch‐and‐wait programmes are now embedded in routine practice, enabling a growing proportion of patients to avoid rectal resection. Trials such as OPRA [1, 2], together with international prospective cohorts [3], have demonstrated durable oncological outcomes in selected responders, shifting definitive surgery from routine to selective.

This evolution offers clear benefits for patients, including reduced morbidity, preserved function and treatment pathways more aligned with quality‐of‐life priorities. For surgical training, however, it presents a challenge. Rectal resection remains a technically demanding procedure with narrow safety margins, yet operative exposure is declining while case complexity is increasing [4]. The resections that do proceed are increasingly weighted toward incomplete responders [5], post‐radiotherapy fibrosis, distorted pelvic planes, threatened margins and salvage after regrowth, scenarios that demand advanced technical judgement and capabilities beyond standard total mesorectal excision (TME) dissection [6, 7]. The index operation has become less frequent and more difficult, while expectations of safety and proficiency remain unchanged.

Traditional training models rely on repeated exposure, progressive autonomy and whole‐case experience over time. These assumptions may become increasingly untenable in low‐volume environments. This Perspective outlines the implications of contemporary rectal cancer treatment for surgical education and proposes practical strategies to safeguard competence in TME and restorative pelvic surgery.

## 1 Clinical Transformation and Its Externalities for Training

The clinical rationale for TNT and organ preservation is well established. Delivering systemic therapy upfront, often combined with radiotherapy, increases the likelihood of tumour regression and enables response‐based treatment decisions. Randomised TNT trials, including RAPIDO, PRODIGE 23 and STELLAR [8, 9, 10], have supported broader adoption of intensified neoadjuvant sequencing and response‐adapted management. However, longer‐term follow‐up from RAPIDO demonstrated higher locoregional failure in the TNT arm [11], underscoring that these strategies are not oncologically equivalent across all endpoints. Structured watch‐and‐wait programmes have matured, with defined imaging, endoscopic and clinical surveillance protocols supporting non‐operative management in carefully selected patients. Local excision may provide an additional organ‐preserving option in selected responders [12].

Together, these approaches have contributed to a measurable reduction in rectal resection rates. In a Dutch national cohort, surgical resection rates for non‐metastatic rectal cancer fell from 85% in 2013 to 73% in 2018 [13]. In the United States, the proportion of patients managed with organ preservation increased from 18.4% in 2006 to 28.2% in 2020 [4]. This reduction in operative volume is accompanied by a shift in case complexity. The resections that remain are more often performed for poor responders [2], locally advanced tumours, or recurrent disease [14] and are frequently post‐radiotherapy, fibrotic or anatomically distorted [15, 16]. The operative learning curve is therefore encountered less often, but is steeper and more variable, with fewer opportunities for graded autonomy. This represents a durable reconfiguration of rectal cancer management.

The issue is therefore not volume reduction alone, but volume reduction coupled with case enrichment for complexity. In practical terms, trainees are exposed to fewer rectal resections, and those they do encounter are less representative of the traditional learning curve.

## 2 Why Rectal Resection Training Is Vulnerable

Technical proficiency in TME requires more than mastery of laparoscopic or robotic colectomy. Rectal cancer surgery demands precise dissection in a confined pelvis, preservation of autonomic nerves, adherence to correct mesorectal planes and reliable achievement of oncological margins [17, 18]. Restorative steps, including distal transection, stapling strategy, anastomotic technique, diversion decisions, and leak mitigation, add further layers of cognitive and technical complexity [19].

The standard training model assumes sufficient volume to permit repeated execution of key steps, exposure to varied pelvic anatomy, and opportunities to encounter and manage common pitfalls. It also assumes progression from assisting to performing substantive components of the operation and ultimately leading the case under supervision. Declining volume disrupts each of these assumptions. Technical progression slows, meaningful autonomy becomes harder to grant and exposure to uncommon but high‐consequence events, such as pelvic haemorrhage or stapler misfire, becomes inconsistent.

Centralisation compounds this challenge. Although higher‐volume centres often demonstrate improved outcomes, centralisation can concentrate operative cases within small groups of surgeons, narrowing the pool of trainers and limiting exposure across a network unless deliberate structures are in place [20, 21]. Inconsistent access to rectal cancer cases risks producing colorectal surgeons with variable capabilities despite meeting time‐based requirements.

## 3 Observable Effects on Exposure, Capability and Workforce Readiness

Exposure is the most immediate consequence of declining operative volume. Trainees may complete rotations with only a small number of opportunities to participate meaningfully in rectal resection [22]. Even when present, they may undertake fragmented components rather than developing whole‐case understanding [23]. Variability between centres widens disparities in experience, with trainees in tertiary units gaining concentrated exposure while others complete training with limited pelvic operating [24]. In a survey of recent US colorectal surgery graduates, 37.1% reported that increasing non‐operative management had negatively affected their preparation for rectal cancer surgery [22]. This is important given that the reported learning curve for minimally invasive TME is commonly in the order of 40 to 80 cases, depending on platform and study methodology [25].

Capability may therefore become more variable [24, 26]. Some trainees progress well due to access to high‐volume pelvic units and committed educators. Others may develop limited familiarity with the operation without sufficient depth across critical steps, particularly beyond TME dissection and anastomotic reconstruction in the deep pelvis [23, 25]. Self‐assessed confidence becomes an unreliable surrogate for competence when experience is limited.

Transition to practice is also affected. Early‐career colorectal surgeons may require prolonged proctoring, co‐surgery arrangements, or stepwise credentialing before performing independent TME. This reflects not inadequate trainees, but a changing training environment in which fewer opportunities exist to consolidate competence before fellowship. Smaller hospitals may face challenges in supporting early consultants who require structured oversight [27, 28].

## 4 Reframing Competence Beyond Case Numbers

In low‐volume environments, procedural counts cannot remain the primary proxy for competence. Numbers remain relevant, but they are necessary rather than sufficient. Competence in rectal resection should encompass technical, cognitive and rescue capabilities. Technical capability includes safe pelvic dissection, identification of correct mesorectal planes, protection of autonomic nerves, deliberate distal transection and reliable anastomosis formation. Cognitive capability includes patient selection, interpretation of staging and response, and intra‐operative decision‐making when planes are abnormal. Rescue capability includes early recognition and management of major intra‐operative or postoperative complications. These domains lend themselves to structured observation, entrustment decisions and progressive assessment, providing a more accurate reflection of readiness for independent practice than raw case numbers.

## 5 Strategies to Support Competence

Several strategies can be implemented within existing training structures. The first is modularisation of TME into discrete, assessable components. Progression should occur stepwise rather than through rare whole‐case autonomy. Modules may include posterior and lateral pelvic dissection, anterior dissection and nerve preservation, distal transection, anastomotic construction and diversion decisions. Each component can be observed and assessed repeatedly, allowing trainees to advance through consistent performance rather than opportunistic participation. This aligns with emerging competency‐based and modular training models in colorectal surgery [29].

Second, simulation should become a routine adjunct. High‐fidelity cadaveric or lab‐based pelvic simulation is ideal, but meaningful training can also be achieved through lower‐cost models, including constrained suturing, stapling practice and crisis drills for pelvic bleeding or difficult transection. Simulation allows rehearsal of infrequent but high‐risk steps, reducing dependence on real case exposure [30, 31].

Third, video‐based coaching provides a scalable method to increase feedback density. Routine recording of key operative phases allows structured review, benchmarking and cross‐site coaching. With appropriate governance, stored video can create a durable repository for training, enabling targeted feedback even when case numbers are low [32, 33].

Fourth, service design should protect training access without defaulting to indiscriminate centralisation. While highly complex pelvic surgery, including multivisceral resection, sacral resection, exenteration and surgery for recurrent disease, may appropriately be concentrated in specialist units, rectal cancer surgery more broadly remains common enough that the priority should be networked access to suitable training cases. Centralisation can improve outcomes in selected settings, but it can also narrow exposure unless paired with deliberate rotational pathways and designated training lists [34].

Finally, structured transition‐to‐practice support is increasingly important. Credentialing processes should reflect the realities of reduced exposure and may include proctoring for early independent TME cases, staged operating privileges, and planned two‐surgeon operating for selected complex cases, particularly in the era of robotic platforms and dual‐console access. Co‐surgery should be viewed as a pragmatic patient‐safety and skills‐consolidation strategy for technically demanding pelvic surgery [35].

Training must also adapt to the competencies required for organ‐preservation pathways. Response assessment, interpretation of MRI and endoscopy, surveillance decision‐making and an understanding of salvage principles are now essential elements of colorectal oncology. Without integrating these skills into training, the shift towards non‐operative management risks creating new capability gaps.

## 6 Conclusion

TNT and organ‐preservation strategies are transforming rectal cancer care, reducing morbidity and aligning treatment with patient priorities. Yet as rectal resections become less frequent and more technically complex, sporadic exposure and opportunistic learning can no longer be relied upon to produce consistent competence in TME. Safeguarding the quality of rectal cancer surgery now requires structured, competency‐based training that evolves in parallel with contemporary practice.

## Data Availability

Data sharing not applicable to this article as no datasets were generated or analysed during the current study.
